# The Missing Professional Perspective: Medical, Veterinary, and Dual Degree Public Health Student Perceptions of One Health

**DOI:** 10.3389/fpubh.2021.704791

**Published:** 2021-07-15

**Authors:** Rohini Roopnarine, Ellen Boeren, Julie-Anne Regan

**Affiliations:** ^1^Department of Public Health and Preventive Medicine, School of Medicine, St. George's University Grenada, Grenada, West Indies; ^2^School of Education, University of Glasgow, Glasgow, United Kingdom; ^3^The Centre for Higher Education Studies, University of Liverpool, Liverpool, United Kingdom

**Keywords:** One Health, role theory, medical, veterinary, dual degree, COVID-19, public health

## Abstract

**Introduction:** One Health (OH) is an important concept to design appropriate public health responses to emerging diseases such as COVID-19. How trainee health professionals understand this concept is important to its implementation. In this study, we explored how medical (MD), veterinary (DVM), and dual degree MD and DVM Master of Public Health (MPH) students define OH and its relevance to practice.

**Methods:** Students participated in a survey that included the Readiness for Interprofessional Learning Scale (RIPLS), and two questions requiring them to define and explain the relevance of OH. The transcripts of the OH responses underwent thematic analysis. Role theory was used to explain the variation in how students from these different programmes viewed the concept.

**Results:** The responses of the MD and DVM students in contrast to the dual degree MPH students reflected gaps in their understanding of the concept that pertained to the specific health impacts of global warming; antimicrobial resistance, food security; social, cultural and environmental determinants of zoonoses occurrence, and health policy formation.

**Discussion:** Mitigation of the global risks to public health require a collaborative approach by health professionals. Our findings suggest that MD and DVM students are unaware of many factors that impact patient health outside of their own discipline. The inclusion of dual degree students revealed novel insights that undertaking an MPH may have enabled them to be more aware about the interdisciplinary relevance of OH to their professional practice. We recommend that structured incorporation of OH should inform future medical and veterinary curricula.

## Introduction

The emergence of the COVID-19 pandemic has highlighted the importance of public health preparedness for mitigating its spread. Increasingly, the current threats to global health posed by antimicrobial resistance, the environmental impacts of climate change, food security, and the emergence of zoonoses such as Ebola and COVID-19, remind us of the interconnection between animal, human, and environmental health. This need for interdisciplinary work within and between the health professions is embodied by the conceptual framework of One Health (OH) (1). Traditionally, interprofessional learning has focused on the shared learning that occurs between students from medicine and the allied human health disciplines, typically excluding veterinarians (2). In an increasingly globalized world, OH—with its public health orientation and embrace of the animal-human-environmental health connection, provides a much needed transdisciplinary framework to address current emerging threats. Settele et al. (3) suggest that OH can facilitate interprofessional collaboration in framing health policy changes that seek to protect the natural environment. Furthermore, Marty and Jones (4) argue the OH framework enables an interdisciplinary approach to be applied to conducting the surveillance necessary in animals, humans and the environment, to identify the likely sources of pandemics such as SARS- CoV-2. This collaborative approach is also important in light of global policy pressures directed by the United Nations as part of its Sustainable Development Goals (5) with a focus on preventing hunger by addressing the issues of food security and climate change.

Successful execution of the OH approach by health professionals requires that they are knowledgeable about the concept. Interprofessional learning has been advocated as a pedagogical tool for enabling the application of the principles of OH to practice, given its core focus on developing competent team workers with excellent communication skills (6). Chapman and Gupta (7) as well as Steele et al. (8) argue that it is crucial for medical students to be familiarized with the principles of OH that connect human, animal, and environmental health to prepare them for their future roles in addressing global health issues, as well as for managing zoonotic infections in their patients. In the same vein, others (9) expressed concerns about the absence of content on ecosystem health within the veterinary curriculum. For example, Lorusso et al. (10) discussed that emerging zoonoses such as COVID-19 indicate that competencies in genomics, social sciences and data management are critical for inclusion within the curriculum for the future veterinarian to actively participate as frontline responders in a public health response. Maeshiro et al. (11) discussed that these competencies also need to be developed as part of the core medical curricula. This is important to avert the gaps in public health preparedness exhibited by the medical fraternity during COVID-19, specifically a lack of focus on preventive medicine, testing and the impact of social disparities on pandemic mortalities.

Within the literature on the need for the introduction of OH in medical curricula, Chapman and Veras (12) discuss that health professional students need to be able to link issues such as antimicrobial resistance, zoonoses and climate change to OH, so that future practitioners have the competencies to address these issues as part of their professional duty to safeguard the population's health. Chapman and Animahasun (13), both medical doctors, also discuss the need for MD students to be exposed to OH early on in their training. This will better prepare them to consider a more holistic approach to arriving at a diagnosis, to communicate health issues with their patients, and to consider the impact of animal and environmental health factors on patient health. Animasahun et al. (14) also discussed that MD students' familiarization with the principles of OH is crucial for their involvement in driving policy change, positively altering human behaviors that negatively impact the environment. For example, they discuss that MD students can use modern technology and social media to educate communities on environmental issues such as the impact of fossil fuels usage on respiratory health and implementing measures to reduce mosquito exposures that lead to vector borne disease emergence.

Decaro et al. (15) discuss that veterinarians with their experience of developing biologics for animal corona viruses can assist future research to develop effective vaccines and antivirals in human medicine for SARS-CoV-2. Similarly, veterinarians should perceive their role in driving policy changes that require animals be kept in hygienic and well-managed market facilities, to mitigate the risk of viral spillover into humans (15). Veterinarians are in a position to drive the OH movement forwards by driving policy changes that mitigate against human invasion of the environmental habitats of wild animals, that contribute to spill-over of diseases from humans to animals. Gibbs and Gibbs (16) also provide excellent examples that portray the role of veterinarians in considering the impacts of ecological health on human and animal health as axiomatic. For example, the effects of use of diclofenac to treat Indian cattle and its devastating effects on decimating scavenging vulture populations had huge implications for ecological health. Human destruction of bat habitats was responsible for the emergence of Nipah virus through human consumption of the amplifier porcine hosts in Malaysia. Gibbs and Gibbs (16) are clear that for these reasons, the North American Veterinary Medical Consortium (NAVMEC) recommend OH knowledge as a key professional competency for veterinarians, and yet it is not an accreditation requirement for medical or veterinary curricula.

The literature to date has in a few instances explored the positive experiences that OH educational interventions have had on MD and DVM students but *not* the students' understanding of OH which is crucial for its execution (17, 18). As we sought to determine how MD and DVM students view the concept of OH, our research question was:

*How do students of the Medical (MD), veterinary (DVM) and dual degree Master of Public Health (MPH) students define the framework of One Health (OH) and its relevance in preparing them for health practice in the global environment?*

The comparison between these groups of students is important as they tend to perceive their future professional roles differently. These students' readiness for interprofessional learning varies according to their perceived roles in the future. Research by the authors of this paper has confirmed that students in a dual degree programme, combining a MD or DVM programme with an MPH are more open to collaborative learning than those who take a stand-alone programme (19). Although, the content pertaining to aspects of OH may vary across MPH curricula at various institutions, MPH students, in contrast to MD and DVM students, are expected to apply their knowledge about the interconnection that exists between animal, human and environmental health embodied by OH on human health (20–22).

## Study Setting

The location of the study is a private medical University, based in the Caribbean. The DVM program is accredited by the American Veterinary Medical Association (AVMA) and the Royal College of Veterinary Surgeons (RCVS); the MPH program by the U.S. Council on Education for Public Health (CEPH) and the MD program by the US National Committee of Foreign Medical Education and Accreditation (NCFMEA). Dual degree DVM MPH and MD MPH students share classes with each other and also with the stand-alone MPH students. Table 1 depicts the portion within each curriculum (credit hours) that includes coverage of topics relevant to One Health such as antimicrobial resistance, zoonoses, foodborne diseases of animal origin, and environmental health.

**Table 1:** MD, DVM, and MPH curricula comparisons: credit hours for One Health topics.

**MD courses**	**Credit hours for One Health topics**	^**^DVM	**Credit hours for One Health topics**	^***^MPH courses credit hours ()	**Credit hours for One Health topics**
Immunology and microbiology (2)	2	Bacteriology (4)	2	Principles of environmental health (3)	3
Public health assessment tools (2)	1	Parasitology (4)	2	One Health: public health applications (3)	3
		Pathology (4)	1	Health policy and management (3)	1
		Virology (3)	2	Social and behavioral aspects of public health (3)	1
		Veterinary public health (2)	2	Practice and leadership of public health (3)	1
		Avian, fish, and exotic diseases (3)	1	Community medicine seminar series (3)	1
		Pathology II (4)	1		
		Small animal medicine (3)	1		
		Livestock medicine (2)	1		
	3 (0.03%)		10.2 (1.5%)		10 (24%)

^*^MD program has a total of 86 credits plus 2 years clinical training.

** *DVM program has a total of 127 credits plus 1 year clinical training*.

*** *MPH program has a total of 42 credits*.

The institution has made very deliberate interventions to make OH a core tenet of its MPH degree. One major change implemented within our MPH degree program was the creation of the mandatory course: “One Health: Public Health Applications.” Thus, not only freestanding MPH students, but all dual degree students are required to take this course. Further, OH issues are being highlighted in many of our other courses, specifically the course on the “Principles of Environmental Health.” Hence, the MPH program can be used as a model for others to follow. Given the focus on OH in the MPH curriculum, it is anticipated that medical (MD) and veterinary (DVM) students pursuing a concurrent MPH may be more aware of the relevance of the interconnection between the sectors embodied by OH, compared to MD and DVM students in standalone programs. Insights from this study will be useful to inform the inclusion of OH content in future curricula.

## Methods

We declare that we obtained Institutional Research Board (IRB) approval for this study. The participants were emailed a copy of the Participant information form 1 week prior to receiving the link to the survey. The survey requiring them to define and explain the relevance of OH, was embedded within the Qualtrics platform. The consent form was also embedded within the Qualtrics platform. Participants indicated their agreement to participate electronically by selecting yes or no, after having read the consent form. Participation was voluntary, and all possible identifiers have been removed to maintain confidentiality of participants in published material.

### Data Collection

The results presented in this paper form part of a larger Mixed Methods Research (MMR) study that was undertaken for a doctoral thesis (23). We invited 864 students in the third year of the MD, DVM program and all dual degree MD MPH and DVM MPH students to participate in a survey. Students invited to participate in the study had to have completed infectious disease courses relevant to understanding OH. The survey was distributed using the Qualtrics platform during October-November 2018. Email reminders were sent to student responders to encourage their participation in the survey. Four hundred and twenty-eight students completed the questionnaire. We then invited faculty and administrators across the schools to participate in focus group interviews to provide their perspectives on the student survey responses. This enabled verification of the themes identified, and minimized any possible researcher bias in the lead researcher's interpretation of the responses, given her role as a veterinarian. The faculty also presented their perceptions of the needs, opportunities and challenges for developing OH in the medical and veterinary curricula, and discussed the development of a vision statement for the University to achieve its execution of its claim to support the One Health philosophy.

The student survey consisted of the Readiness for Interprofessional Learning Scale (RIPLS) (24) and asked students if they are familiar with the term OH. If so, two open-ended follow-up questions were asked with the following instructions: (1) How do you define the concept of OH? and (2) What do you consider the relevance of the OH concept might be to your practice as a global health professional? Additional questions required students to provide demographic data pertaining to their age, gender, ethnicity, nationality, program of enrolment, prior public health experience, and familiarity with OH. Table 2 depicts the student demographics.

**Table 2:** Demographics of student participants.

**Variable**		**MD**	**DVM**	**MD MPH**	**DVM MPH**	**Total**
Gender (428)	Male	99	11	50	<5	164
	Female	133	65	43	15	256
	I prefer not to say	<5	<5	<5	<5	7
	Other	<5	<5	<5	<5	<5
Ethnicity (428)	Black/African American	40	<5	12	<5	56
	Asian	63	<5	33	<5	101
	Hispanic or Latino	20	7	6	<5	34
	White	78	58	25	16	177
	Native American	<5	<5	<5	<5	<5
	Other	36	5	18	<5	59
Age Range (428)	18–24	59	21	15	9	104
	25–34	168	56	72	9	305
	>35	10	<5	7	<5	19
Nationality (428)	US/American	176	69	62	17	324
	Canadian	13	6	11	<5	30
	Asian	7	<5	5	<5	13
	Caribbean	23	<5	10	<5	33
	African	10	<5	3	<5	13
	Other	<5	<5	<5	<5	8
	British	<5	<5	<5	<5	<5
	European	<5	<5	<5	<5	<5
Total by Program		237	78	94	19	428

The analysis of the student responses from the RIPLS questionnaire now form part of a recently published paper by the authors (19). Student responses on the definition of OH and its relevance to practice are the focus of the present article.

### Data Analysis

The researchers used NVivo version 12 software as a tool for organizing the data from the student responses into case files at case nodes according to the programs represented in this research (DVM, MD, MD MPH, DVM MPH). Braun and Clarke's (25) six step process was used to conduct thematic analysis on the participant responses pertaining to OH. Themes and subthemes were identified emerging from the student responses through inductive reasoning.

To interpret the findings of this study, we used the structuralist lens of Role theory (26) which proposes that professional role expectations are influenced by the individual's institutional context and the culture of their professional program. As mentioned above, DVM, MD, and dual degree MPH students have different expectations of their professional role, which is expected to influence their views about the meaning and relevance of One Health, and by extension collaborative practice.

## Results

The results presented in this paper follow on from findings presented by Roopnarine and Boeren (19) elsewhere. Based on an analysis of the students' scores from the RIPLS (24), it became clear that students undertaking an MPH are more ready for learning cooperatively with other medical students and by extension collaborative practice. These results highlighted the need to further invest in the development of Communities of Practices (27) to encourage MD and DVM students and faculty to engage in shared projects and learning events. Underpinned by the philosophy of OH, this might then lead to an established culture of collaborative learning and the development of an interprofessional identity among all students, not just MPH students. Together with the focus on “readiness for interprofessional learning,” this study also investigated students' familiarity and understanding of the OH concept. It is this part of the research that is being presented in this paper.

Of the 428 students that participate in the survey, 322 students (75 percent) indicated their familiarity with the term “One Health.” Among those 322 students, 265 students wrote down a definition of One Health (121 MD students; 67 DVM students; 61 MD MPH students, and 16 DVM MPH students) and 273 students (122 MD students; 72 DVM students; 62 MD MPH students, and 17 DVM MPH students) described the relevance of OH to their future practice.

Outcomes of the analysis of definitions and the understanding of the relevance of OH as provided by students will be presented below. Within each theme we first describe how students defined the concept of OH generally, and then focus on how they define the relevance of OH as it pertains to their future professional practice. Three themes will be discussed and will expand upon the students' perceptions on the linkages between human and animal health and the role of environmental issues in a globalized world. As will be evident from the sections below, students from different programmes vary in the importance they attribute to human, animal, and environmental aspects in discussing One Health.

### Theme 1: Interprofessional Collaboration and Zoonoses Prevention

The participant responses to the questions requiring them to define the OH concept and its perceived relevance to their future practice that support this theme, are shown here in Table 3.

**Table 3:** Interprofessional Collaboration and zoonoses prevention.

**Questions**	**Participant responses**
Definition of One Health	*DVM student: Medical professionals working together to improve the health and well-being of humans and animals*. *DVM student: Being able to allow veterinary medicine and human medicine to interact together with a common goal and protect everyone against zoonotic diseases*. *MD student: The concept of interdisciplinary cooperation and degrees of integration between the fields of Medicine and Veterinary Medicine driven by the significant impact of each field on the other, and the need to enhance effectiveness in the prevention and treatment aspects of patient care on individual and societal levels*. *MD student: Cooperation in research & practice between MDs and DVMs* *MD MPH student: “Physicians, Veterinarians and public health professionals working together to educate patients and practice preventive medicine to improve the health of all species.”* *MD MPH student: One health is an approach to implementing policies and legislation in which multiple sectors work together to achieve a better health outcome. These may include preventing spread of infectious diseases and combating antibiotic resistance*.
Relevance of One Health	*DVM student: MDs and DVMs being able to work together when there is an outbreak to figure out the best way to control it*. *DVM student: Veterinarians are an important resource for pet owners, farmers, …to receive information regarding zoonoses…veterinarians may also be a useful resource for medical practitioners in quickly identifying such infectious diseases with which the practitioner may be less familiar*. *MD student: As a medical professional focusing on health and human illness, I must focus on zoonoses. If I pay attention to how an animal's life may be affecting my patients, I may be more likely to recognize a zoonotic disease if present*. *MD MPH student: Maintaining global relationships with Vets, engineers, physicians, government, and corporate officials to address important global health issues*.

Across the different programs, students predominantly defined the concept of OH as representing the collaborative partnership between veterinarians and medical doctors. However, there were distinct disciplinary differences, with MD students far less likely to mention partnership with veterinarians. DVM students, on the other hand, were very clear about the human-animal link embedded in the OH concept and the need for collaboration with medical doctors.

For the MD students, human health emerged as the key theme associated with the concept of OH, although, the occasional MD student saw the collaboration between human and veterinary medicine as key to the concept. DVM students tended to define this collaborative partnership between medical doctors and veterinarians with a stronger focus on protecting the public from zoonotic diseases.

There is a clear link between zoonosis and OH for DVM students, which highlights for them the collaboration necessary across the human-animal boundary. This cross-boundary definition was also apparent with the dual degree students. However, the dual degree students defined the concept with a more embracing type of collaboration, involving a wider group of health professionals than the MD and DVM students. The dual degree students often saw the relevance of the concept as embracing collaboration to optimize the health of all species by collaborating at the “*multisectoral”* level using “*transdisciplinary*” approaches to solving issues and tackling these at the “*local, regional and global levels*.” They also frequently included the notions of prevention and education within their definitions of OH.

Unlike the dual degree students, few MD and DVM students defined OH in association with health policy formation, although, some viewed OH as including“*epidemiological tracking of disease”* along with measuring the “*social/economic impact of disease in which policy is key.”*

On reflecting on why there were differences in how students perceived the type of interprofessional collaboration that defined OH, we identified a number of factors. Medical students expect to work with nurses and other allied human health professionals in their clinical rotations and then to see interprofessional practice as collaborations between these groups of practitioners. MPH students undertake courses on health policy formation that expose them to the role of government officials in driving policy changes. Veterinarians are focused on zoonoses prevention as crucial to their role in protecting public health and thus this may explain their focus on collaboration with medical doctors for this purpose.

In discussing the relevance of OH to practice, students differentiated on the emphasis placed on zoonoses in the collaboration between MDs and DVMs. Although, MDs were clearly aware of the relevance of OH for preventing zoonoses occurrence in their patients, DVM and DVM MPH students felt greater responsibility to prevent zoonoses in animals as key to their role in educating and working with the medical doctor to protect the public's health. For the veterinarians, the relevance of OH to their practice was tied into the vets' role in educating the medical doctor on the impact of zoonoses on human health. While DVM students frequently mentioned specific zoonoses of concern such as “*Ebola,” and “rabies”* it was noticeable that for the MDs, examples of specific zoonoses of global health importance were omitted in their responses about the relevance of OH.

In contrast to the MD and DVM students, dual degree students perceived the relevance of OH to practice through its inclusion of government officials and public health workers in these collaborative efforts for driving health policy changes. This relevance of OH, outside of direct patient care (animal or human) found in dual degree students perhaps reflects why some chose to enroll on the dual degree. Their views of possible future practitioners is not confined to direct patient care as was commonly found with single MD or DVM students.

### Theme 2: Incorporation of Environmental Health and the Expanded Area of Climate

The participant responses to the questions requiring them to define the OH concept and its perceived relevance to their future practice that support this theme are shown here in Table 4.

**Table 4:** Incorporation of environmental health and the expanded area of climate.

**Questions**	**Participant responses**
Definition of One Health	*MD MPH student: One health means that we are focusing on all aspects of our environment—humans, animals, all aspects of the living environment and human action (factories, etc.,)—in our definition of what affects our health* *MD student: Concept that the health of human beings is connected to animals and the environment*. *MD student: One Health describes the importance of global health…how air, water, sanitation, environment, and climate change impact global health*. *DVM MPH student: “The interweaving of animal, human and environmental health and its overall impact on the welfare and well-being of the global community*. *DVM MPH student: One Health is based on the principle that humans, animals, and the environment share a web of interconnections and the health status of any one of these affects the others. One Health is achieved when the highest level of health in humans, animals and the environment is reached*.
Relevance of One Health	MD MPH student …* Furthermore, human activities have caused significant damage to the environment and natural ecosystems, which in turn could have devastating consequences to human, animal, and plant health ….public health challenges we face include pollution of the atmosphere …., deforestation, …… urban expansion and poor land management, ……, loss of biodiversity and ……, and climate change/global warming*. *DVM MPH student: Not only will my job be to protect and treat animals, it will also be my duty to protect humans by controlling zoonoses transmission. This will also involve protecting and promoting environmental health by working to reduce harmful emissions and combat climate change to further reduce the prevalence of vectors that spread zoonotic diseases*. *MD student: We need to be aware of the factors that impact patient care outside of their control: environment, economic, social, political*. *MD student: To have a better understanding of how the environment that my patients are in, and zoonotic factors, can affect their health*.

The second theme identified, in terms of defining OH and discussing its relevance, reflects the inclusion of environmental health and potential threats. In defining OH, again there were distinct disciplinary differences noted. While some MD students recognized the role of the environment in the definition of OH, the environment was clearly omitted by most DVM students. However, the dual degree students were all clear on the role of the environment within the concept of OH. Some MD students defined the role of the environment within the concept as synergistic with human and animal health. While others saw the role of environmental health only as it was relevant to human health.

With regards to how students perceived the relevance of OH to their future practice, it was clear that for the dual degree students, the role of environmental factors was key. Specifically, they saw OH as relevant for managing the impact of “*biodiversity loss,” “urbanization,” “climate change and global warming,” “air and water pollution”* and the occurrence of “*vector borne diseases”* on human and animal health.

When discussing the relevance to their future practice, the disciplinary differences continue as might be expected. The dual degree students clearly see a role for themselves in mitigating against the effects of environmental hazards on human and animal health, therefore, the concept of OH will have relevance to them in that role. Whilst a number of MD students included environmental factors in the definition of OH, they did not necessarily see that as being relevant to their future practice. In other words, although, they recognized the impact of the environment on human health, their role was not to manage those environmental factors, therefore, that aspect of OH was not seen as relevant to their practice. This links back to their lack of insight into a possible role in influencing health policy and other public health measures. However, where the environment was recognized as part of OH, they did it as relevant in the care of their patients. The notable absence of environmental health in DVM students' definition of OH was understandably reflected in their views on its relevance, marking a stark difference to their DVM peers undertaking an MPH degree.

### Theme 3: Human Health and the Expanded Areas of Antimicrobial Resistance, Human-Animal Bond and Food Systems

The participant responses to the questions requiring them to define the OH concept and its perceived relevance to their future practice that support this theme are shown here in Table 5.

**Table 5:** Human health and the expanded areas of antimicrobial resistance, human-animal bond and food systems.

**Questions**	**Participant responses**
Definition of One Health	*MD student: the integration of medical aspects, and various professions that focus on the well-being and health of the public.”* *MD student: One Health is an organization that wants to provide the best healthcare to people across the globe* *MD student: One Health means to combine all forms of healthcare and health education to provide quality care to all individuals, It not only focuses on screening, but on patient education. This allows for development of sustainable care*. *MD student: The interpersonal relationship between physicians, patients, and researchers to improve medicine*. *MD student: One Health to me is treating the whole person, not just the illness. It means asking not only about physical signs of illness, but also about what may be affecting their mental and emotional state*.
Relevance of One Health	*DVM: It is extremely important to understand the ways in which drug residues can affect consumers. As a food animal vet it will be my job to ensure the safety of food that many people consume on a daily basis.”* *DVM student: Healthy animals have also been linked to decreasing depression, and other mental illnesses …*. *DVM student: Zoonoses, antibiotic resistance, pandemics-its important to work with MDs to prevent them*. *MD MPH student: Encompassing various aspects of population health from prevention to social interactions so a holistic approach is achieved*.

Most MD students interpreted OH as solely defining the improvement of access to healthcare for PEOPLE, regardless of age, ethnicity and gender. What was specifically absent in the responses from MD students was an awareness of the role of animal health or to a lesser degree, environmental health. Their definitions of OH were predominantly indicating an approach to health for all humans, with very little understanding of the interconnection between animal, human, and environmental health. Their views included the need to provide optimal programs for healthcare, to considering the mental as well as physical health of their patients, in addition to other social and economic determinants of health.

The expanded issues referred to in this theme, antimicrobial resistance, food safety and the human-animal bond, were only apparent in the responses of the DVM students responding to the question about the perceived relevance of OH to practice. DVM students associated OH with their key professional role to protect the public health from issues such as antimicrobial resistance and foodborne diseases of animal origin. DVM students mentioned their role to consider the safety of “*human workers….and other consumers”* and their role in foodborne disease “*outbreaks.”* A number of DVM students highlighted the importance of the animal-human bond as beneficial for optimizing human mental health. DVMs were clearly aware of the link between animal and human health promotion and OH was viewed as relevant to that aspect of their practice.

Generally, across the themes identified above, responses indicate that students across all programs perceived the relevance of OH to their future practice. The way they view this responsibility differs across the programmes, but they all see it as relevant to their contribution to health issues in a global world. In general, the dual degree students have a more inclusive definition of OH and their envisaged role is more likely to encompass human, animal, and environmental health. In addition, they view their future role more broadly than treating their human or animal patients, envisaging a role in developing and influencing public health policy, which the single degree students did not express in this study. As part of this MMR study, the faculty discussions led to the creation of a vision statement shown in Table 6, for the University to provide an expectation to future student recruits across the disciplines about the type of medical education they will be receiving.

**Table 6:** Vision for the Institution.

*Health will be perceived as the embodiment of a shared experience involving the preservation of animal, human, and environmental health for optimizing the health of all species and the environment they live in. Thus, it is the vision of this institution that medical and veterinary education will support and advance global health in its educational, research, and social activities within the framework and realization of the One Health concept. Shared interprofessional learning opportunities across the disciplines will promote the development of graduates that will transform healthcare through a collaborative approach to practice*.

## Discussion

As discussed at the start of this paper, it is important for future medical practitioners to be familiar with the concept of OH in order to deal with urgent medical issues. However, not all medical curricula pay significant attention to OH, resulting in insufficient understanding of this concept among the student population. These gaps in the curriculum seem to relate to the impacts of global warming on human and animal health; food security concerns; the impact of socio-cultural and environmental factors on the occurrence of zoonoses; the role of the human-animal bond on human mental health; the role of the MD and DVM in response to pandemics such as SARS-CoV-2, health policy development and the implications of antimicrobial resistance for public health.

Work by Chapman and various of her colleagues, as discussed above, indicated a need for health professional students to be prepared for collaborative practice in the future and to better understand the interactions between human, animal, and environmental health spheres (7, 12). Amihasun et al. (14) paid specific attention to the need for a more holistic approach to diagnoses to be developed among MD students as this would help them to better consider the impact of animal and environmental factors on their patients' health. Decaro et al.'s (15) focus on veterinarians needing to step up to be at the forefront of policy changes during pandemics such as COVID-19 highlighted another need for introducing students to OH. However, our data show that not all students in our study demonstrated high levels of awareness of these issues, hinting at significant gaps in the university's curricula.

Through the inclusion of the dual degree MPH students in this study, evidence arose that the MPH curriculum is closing some of these knowledge gaps for MD and DVM students who are enrolled in the dual degree programme. As introduced above, the introduction of OH is more prominent in the MPH curriculum than in the standalone MD and DVM programmes and these dual students are thus more aware of the need to work along the human, animal, and environmental spheres of health issues.

Exploring the relationship between human and animal health, Rabinowitz and Conti (28) discussed that medical doctors are expected to advise clients on proper hygiene when handling pets and referring clients to a veterinarian to choose an appropriate pet, recognizing the mental health benefits provided by human-animal bonding. Most of the MD respondents however did not mention these aspects of the concept of OH. The DVM and DVM MPH in contrast to the MD students were aware of the risks of two critical issues to human health: food security issues and antimicrobial resistance.

Specifically in relation to the interface between animal and human health on the one hand and environmental health at the other hand, the awareness of health factors affected by globalization are especially important in the current day context of a pandemic (29). Addressing global zoonotic diseases like COVID-19 that impact human and animal health, requires collaborative OH approaches by social, public health, environmental, medical, veterinary, and social care professionals (30–32). Hence, MD and DVM students must be aware of the social, economic, and epidemiological disease determinants that lead to zoonotic diseases that are relevant to international health practice (33). It was clear from our data that MD MPH and DVM MPH students demonstrated a more sophisticated understanding of OH and its relevance for their own professional practice compared to MD and DVM students in the standalone programmes. MD and DVM students seemed unaware of the relevance of key global issues such as climate chance to OH and by extension to their future roles in clinical practice. While DVM students frequently mentioned specific zoonoses of concern it was noticeable that MDs students omitted these reflections on the link between zoonoses and global health as part of their responses to questions about OH. None of the MDs mentioned specific zoonoses of global relevance which is consistent with Rabinowitz and Conti's (28) observation that medical curricula tend to place little emphasis on zoonoses and even less any mentioning of OH content. MD students also seemed unaware of the impact of global warming on the rise of vector borne diseases, natural disasters, the occurrence of heatstroke and respiratory diseases and allergies on their patients. This is a worrying result as Chapman and Dunham (34), discuss that zoonoses and antimicrobial resistance “exemplify” (p. 332) OH. Chapman and Dunham (34) discuss that increasingly, environmental health, food security, antimicrobial resistance should be incorporated within medical curricula as essential OH competencies. They argue such competencies are necessary to address global threats by stakeholders across the nexus of animal, human and environmental health. The occurrence of natural disasters resulting from climatic change (35) coupled with transboundary diseases in animals subsequent to global trade, has compromised agricultural production globally (36). The latter has led to a reduction in food availability and food security affecting 820 million persons globally, reflecting the urgent need to build partnerships to achieve these goals (36).

DVM students often omitted to consider the role of the environment as relevant to their future practice in animal health, even though climate change and global warming are known to have implications for heat stroke in animals. There was no consideration of the influence of social and behavioral or cultural factors on zoonoses occurrence by the DVM respondents. Given the current climate of global health, it was surprising that DVM students did not mention the behavioral factors such as bushmeat consumption on Ebola occurrence or the spread of highly pathogenic H5N1 avian influenza viruses causes by close contact between humans and domestic poultry in rural global environments. Importantly, there was little mention of the impact of global warming on the spread of vector borne diseases, or the role that human destruction of wildlife habitats has on placing humans at greater risk of zoonoses exposure through increased contact with wildlife reservoirs. It seems in this sample, that DVM students did not consider these issues will be relevant to their future practice as veterinarians. These findings may be partly explained by the fact that while DVM students are exposed to discussions on the impacts of climate change and socio-cultural factors on zoonoses occurrence in their veterinary public health course, environmental impacts on animal health are not discussed in the DVM curriculum.

Having identified gaps in the current MD and DVM understanding of OH and its relevance, we examine how those gaps can be addressed in the curricula. We agree with the observation by Rabinowitz and Conti (28) that with OH the lack of coverage of environmental health in the MD and DVM curricula is an issue, and that this will have a knock- on effect on how future practitioners will be able to deal with global disease threats. Food security, adequate water and air quality should be discussed as key to preventing the occurrence of infectious diseases (28). In the MD curriculum, there is also the need to directly address the connection between animal and human health, and raise awareness of the interprofessional collaboration across that boundary (6). While dual degree students identified all of these areas as key to the relevance of OH and to their public health responsibilities as future practitioners, these aspects also need to be integrated in the MD and DVM curricula.

Focussing on the DVM curriculum in particular, it is recommended that students should receive exposure to more structured OH content that brings in environmental health content that enables them to be aware of their role in mitigating the impacts of wildlife habitat destruction that has implications for both loss of biodiversity as well as zoonoses emergence (37). DVM students should have curricula content exposure that promotes their awareness of their role in mitigating the impacts of global warming and other environmental hazards (9, 37). Disease concepts relevant to ecosystem health and environmental perspectives on aspects of herd management could be considered within DVM curricula. As Stephen (9) discusses, mitigating the impacts of outbreaks such as foot and mouth disease would require students to have the skills required to engage multiple stakeholders in formulating policies to deal with mass animal destruction and carcass disposal, and for addressing the impacts of these outbreaks on communities. Kaufman et al. (38) discuss how Tufts University in the US, has integrated core principles of ecosystem health across both its 4-year core curriculum as well as through elective opportunities for students with a special interest in the field of conservation medicine.

As introduced above, role theory (26) help understanding our findings too. MD and DVM students construct their imagined futures as practitioners in a specific way. This often includes working in silos from each other. As Rabinowitz and Conti (28) discussed, MDs are aware of their role in treating zoonoses in their patients but do not tend to do this in collaboration with veterinarians. As Steele et al. (8) argued, students need to be made more aware of their joint responsibilities in tackling diseases, underpinned by a stronger need for communication between diverse health professionals, more engagement in teamwork, and a better understanding of each other's specific roles. Raising this awareness should ideally start at university. As discussed by Steele et al. (8), Gibbs and Gibbs (16), and Rabinowitz and Conti (28), professional roles and expectations should be clearly articulated to future OH veterinarians and medical doctors as part of the curriculum. Through the inclusion of the dual degree students, this study provides a novel insight into how the content of an MPH curriculum closes gaps on specific environmental health knowledge that is evident within the MD and DVM programmes. For the DVM MPH and MD MPH students, the role of the environment in the OH concept was recognized as necessary, suggesting that there is an expectation among these students to link environmental health content to their practice. This view is supported by others (11) who discuss the benefits of public health education, proposing that public health education enables students to consider the multifactorial causes of disease, thus promoting efforts to minimize climate change.

## Conclusion

The study was unique in that it provided novel insight through the inclusion of dual degree MPH students alongside MD and DVM students for illustrating the advantage of familiarizing MD and DVM students with the principles of OH for practice. The study also used the lens of role theory for showing the relevance of OH and by extension students' perceptions on collaborative practice within their future professional roles. A limitation of this study is that we relied on student responses from open-ended questions on an online survey which did not provide the opportunity to seek further clarification from anonymous student respondents. However, the anonymity was designed to reassure respondents that they could express their views freely without fear of being “wrong.” Research findings are limited to one institution only, so contextual issues may strongly influence findings. However, the findings seem to be largely in line with studies elsewhere, which is reassuring.

The comparative perspectives provided in this study, specifically by including dual degree students exposed to OH through the MPH have significant implications for recommending curriculum changes toward incorporating content on OH as fundamental to producing globally ready medical doctors and veterinarians (39). The MPH program and its related content can provide guidance for how OH may be successfully integrated and delivered within these core programs.

In this study, the authors did not seek to change the role definition for the future medical doctor and veterinarian nor to increase the curriculum workload. Rather the findings of this study demonstrate that having an awareness of the multiplicity of causal factors can lead to a better understanding of how complex health issues can be addressed. For too long, efforts to manage infectious diseases such as Highly Pathogenic Avian Influenza (HPAI), Ebola and now COVID-19 by international health agencies has failed. The zoonotic and environmental origins of these deadly disease threats adds significant weight to the argument for the need to prepare future health professionals to act to mount an effectual public health response to these threats, necessitating the inclusion of OH in the curricula of the MD and DVM programmes (40).

## Data Availability Statement

The original contributions presented in the study are included in the article/supplementary material, further inquiries can be directed to the corresponding author/s.

## Ethics Statement

The studies involving human participants were reviewed and approved by St George's University Institutional Research Board (IRB) and the University of Liverpool Virtual Programs Research Ethics Committee (VPREC). The patients/participants provided their written informed consent to participate in this study.

## Author Contributions

RR was the principal investigator and provided the conceptual framework for the study and conducted the research. J-AR and EB were the doctoral supervisors for the principal investigator.

## Conflict of Interest

The authors declare that the research was conducted in the absence of any commercial or financial relationships that could be construed as a potential conflict of interest. The reviewer BK declared a past collaboration with one of the authors RR.
